# Anti-Inflammatory Effects of Traditional Chinese Medicines against Ischemic Injury in In Vivo Models of Cerebral Ischemia

**DOI:** 10.1155/2016/5739434

**Published:** 2016-09-15

**Authors:** Chin-Yi Cheng, Yu-Chen Lee

**Affiliations:** ^1^School of Chinese Medicine, College of Chinese Medicine, China Medical University, Taichung 40402, Taiwan; ^2^Department of Chinese Medicine, Hui-Sheng Hospital, Taichung 42056, Taiwan; ^3^Department of Chinese Medicine, China Medical University Hospital, Taichung 40447, Taiwan; ^4^Research Center for Chinese Medicine & Acupuncture, China Medical University, Taichung 40402, Taiwan; ^5^Graduate Institute of Acupuncture Science, China Medical University, Taichung 40402, Taiwan

## Abstract

Inflammation plays a crucial role in the pathophysiology of acute ischemic stroke. In the ischemic cascade, resident microglia are rapidly activated in the brain parenchyma and subsequently trigger inflammatory mediator release, which facilitates leukocyte-endothelial cell interactions in inflammation. Activated leukocytes invade the endothelial cell junctions and destroy the blood-brain barrier integrity, leading to brain edema. Toll-like receptors (TLRs) stimulation in microglia/macrophages through the activation of intercellular signaling pathways secretes various proinflammatory cytokines and enzymes and then aggravates cerebral ischemic injury. The secreted cytokines activate the proinflammatory transcription factors, which subsequently regulate cytokine expression, leading to the amplification of the inflammatory response and exacerbation of the secondary brain injury. Traditional Chinese medicines (TCMs), including TCM-derived active compounds, Chinese herbs, and TCM formulations, exert neuroprotective effects against inflammatory responses by downregulating the following: ischemia-induced microglial activation, microglia/macrophage-mediated cytokine production, proinflammatory enzyme production, intercellular adhesion molecule-1, matrix metalloproteinases, TLR expression, and deleterious transcription factor activation. TCMs also aid in upregulating anti-inflammatory cytokine expression and neuroprotective transcription factor activation in the ischemic lesion in the inflammatory cascade during the acute phase of cerebral ischemia. Thus, TCMs exert potent anti-inflammatory properties in ischemic stroke and warrant further investigation.

## 1. Introduction

Stroke is the third leading cause of death in developed countries [1] and the major cause of severe long-term disability worldwide [1–3]. Approximately 15 million people experience stroke annually. Of these, one-third die and one-third experience permanent disabilities, thus imposing considerable social and economic burden [4]. Approximately 80%–85% of all stroke events are ischemic caused by cerebral arterial thrombosis or embolism [5, 6]. To date, recombinant tissue plasminogen activator (rtPA) is the only Food and Drug Administration-approved medical therapy for acute ischemic stroke. However, rtPA has severe disadvantages, including the narrow therapeutic time window of 4.5 h and potential risk of hemorrhagic transformation; therefore, the eligibility of rtPA is reduced to only 4%–7% in all the patients with acute ischemic stroke [5]. Thus, potential therapeutic strategies for ischemic stroke are urgently needed.

Increasing evidence has demonstrated that inflammation plays a pivotal role in the pathophysiology of acute ischemic stroke [3, 5, 7]. During acute ischemic stroke, the brain is injured by ischemia- and inflammation-related primary and secondary insults [5]. The primary injury occurs at the beginning of ischemia; it rapidly interrupts the cerebral blood flow to the ischemic core and subsequently causes a significant decrease in oxygen and glucose supply to cerebral neurons [8, 9]. The secondary injury is attributed to the postischemic inflammatory cascade, which produces various proinflammatory mediators, including cytokines, chemokines, proteases, and cell adhesion molecules, leading to an exacerbated ischemic brain injury [10]. However, the postischemic inflammatory response has a disadvantage and an advantage, exacerbating ischemic brain damage in the early phase and triggering tissue regeneration in the delayed phase, respectively [1, 2].

The lack of effective and widely applicable therapeutic strategies for the treatment of ischemic stroke has triggered increasing interest in traditional medicines, particularly traditional Chinese medicine (TCM) [11, 12]. Several centuries ago, TCM was used in China to treat cerebrovascular disorders, including stroke. Evidence revealed that TCM preparation, Chinese herb medicine, and TCM-derived active compounds exert anti-inflammatory effects by inhibiting inflammatory mediators, leukocyte infiltration, and blood-brain barrier (BBB) disruption in experimental cerebral ischemia [13]. These potent effects of TCMs against cerebral ischemic injury highlight their potential in clinical applications. Therefore, this review summarized the origin and development of the postischemic inflammatory cascade and delineated the anti-inflammatory effects of TCMs (namely, TCM-derived active compounds, Chinese herbs, and TCM formulations) on the basis of the in vivo literature.

## 2. TCM-Mediated Downregulation of Microglial Activation

### 2.1. Activation of Microglia in the Initial Phase of Cerebral Ischemia

In the acute phase (min to h) of cerebral ischemia, ischemic injury triggers a rapid activation of resident microglia in the brain parenchyma [3, 14]. During cerebral ischemia, microglial morphology changes from a ramified to an amoeboid shape upon activation [15]. In the initial stage of ischemia, the injured neurons expose damage-associated molecular patterns (DAMPs), which are subsequently recognized by toll-like receptors (TLRs), such as TLR4, and other pattern recognition receptors on the surface of the reactive microglia; this recognition triggers microglia-mediated inflammatory mediators release, contributing to secondary damage after stroke [6, 10, 16]. Reactive microglia/macrophages can be detected as early as 2 h after cerebral ischemia and maintained up to 1 week after the ischemic insult [6]. Reactive microglia are divided into two phenotypes: the classically and alternatively activated phenotypes (M1 and M2, resp.) [17]. The M1 microglia produce proinflammatory mediators, such as cytokines [interleukin- (IL-) 1*β*, IL-6, IL-18, and tumor necrosis factor- (TNF-) *α*], chemokines [monocyte chemoattractant protein- (MCP-) 1, and macrophage inflammatory protein- (MIP-) 1*α*], interferon- (IFN-) *γ*, matrix metalloproteinase- (MMP-) 9, and reactive oxygen species (ROS) [18], exerting detrimental functions in the early phase. By contrast, the M2 microglia secrete anti-inflammatory mediators, such as IL-4, IL-10, IL-13, transforming growth factor- (TGF-) *β*, and insulin growth factor-1, exerting neuroprotective effects in the delayed phase [2, 3, 19]. In cerebral ischemia, microglial activation is accompanied by reactive astrogliosis, which also produces an excessive amount of cytokines and causes the exacerbation of ischemic brain injury [18].

### 2.2. The Effects and Mechanisms of TCMs on Inhibiting Microglial Activation in In Vivo Models of Cerebral Ischemia

Hsieh et al. reported that Paeonol, a common compound of* Paeonia suffruticosa* Andrews (Chinese name, Mu Dan Pi; Moutan cortex), reduces cerebral infarct and neurological deficits at 1.5 h of ischemia and 24 h of reperfusion. Paeonol exerts anti-infarct effect mainly by inhibiting microglial activation and IL-1*β* expression in the ischemic cortex in ischemia/reperfusion- (I/R-) injured rats [20]. Pretreatment with tetramethylpyrazine (TMP), an active compound isolated from* Ligusticum wallichii* Franch (Chuan Xiong), effectively reduces the cerebral volume by inhibiting myeloperoxidase (an inflammation marker) and ED1 (a microglia/macrophage marker) expression in the ischemic core 72 h after reperfusion. The effect of TMP against microglial activation-mediated neurotoxicity can be further attributed to the suppression of prostaglandin E2 (PGE2) in the ischemic core [21]. A later study reported that TMP provides neuroprotection against ischemic brain injury partially by inhibiting microglial activation and subsequently downregulating MCP-1 expression in the ischemic cortex 72 h after reperfusion [22]. Andrographolide, the major active compound derived from* Andrographis paniculata* (Chuan Xin Lian) protects against cerebral infarction and ameliorates neurological deficits 24 h after permanent middle cerebral artery occlusion (MCAo). Andrographolide exerts neuroprotective effects partially by inhibiting microglial activation and microglia-mediated IL-1*β* and TNF-*α* expression in the ischemic area [23]. Pretreatment with* Sophora japonica* L. (Huai Hua; intraperitoneal injection) effectively reduced the cerebral infarct area and neurological deficits at 1.5 h of ischemia and 24 h of reperfusion. The effects of* S. japonica* involve the suppression of microglial activation and microglia-mediated IL-1*β* expression in the ischemic cortex [24]. Lim et al. demonstrated that an intragastric administration of total isoflavones isolated from* Pueraria lobata* (Ge Gen; TIPL) significantly reduces the cerebral infarct volume at 2 h of ischemia and 48 h of reperfusion. The anti-inflammatory effect of TIPL is partially attributed to the inhibition of astrocyte and microglial activation in the hippocampal CA1 region 7 d after MCAo [25].

**Figure 1:**
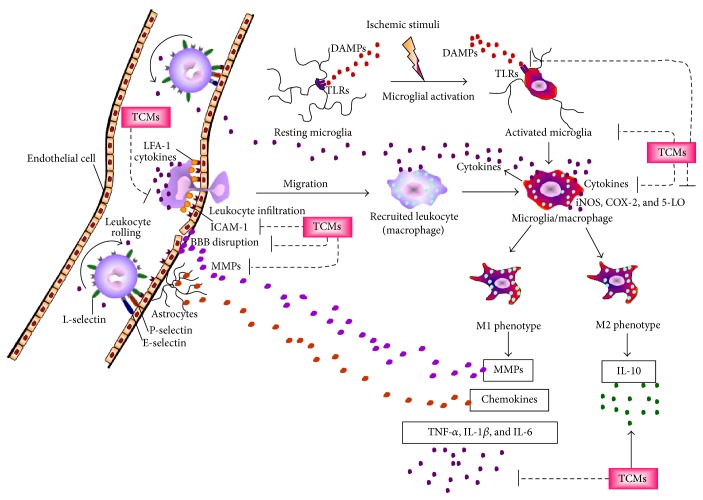
Schematic representation of the effects of traditional Chinese medicines on inflammation responses in the inflammatory cascade after cerebral ischemia. TCMs, traditional Chinese medicines; DAMPs, damage-associated molecular patterns; TLRs, toll-like receptors; LFA-1, leukocyte function-associated antigen-1 (CD11a/CD18); ICAM-1, intercellular adhesion molecule-1; MMPs, matrix metalloproteinases; iNOS, inducible nitric oxide synthase; COX-2, cyclooxygenase-2; 5-LO, 5-lipoxygenase; BBB, blood-brain barrier. Thick solid lines with arrowheads indicate activation, and thin dotted lines indicate inhibition.

On the basis of these studies, the anti-inflammatory effects of TCMs against cerebral ischemic injury could be attributed to the downregulation of microglial activation and microglia-mediated proinflammatory cytokines production in the ischemic area during the initial phase of MCAo (Figure 1 and Table 1).

## 3. TCM-Mediated Suppression of Leukocyte Infiltration

### 3.1. The Process of Leukocyte Infiltration during Cerebral Ischemia

The microglial and astrocytic production of proinflammatory mediators rapidly increase the expression of adhesion molecules on the endothelium [26, 27]. During acute cerebral I/R injury, the peripheral leukocytes first roll, become activated, and consequentially attach to the endothelial cells in the ischemic lesion. Leukocyte-endothelial cell interactions in inflammation are mediated by various adhesion molecules, including selectins, integrins, intercellular adhesion molecule- (ICAM-) 1, and vascular cell adhesion molecule-1 [28]. Selectins, comprising L-selectin on leukocytes and E- and P-selectins on endothelial cells, are a family of lectin-like adhesion glycoproteins that regulate leukocyte rolling and recruitment [28, 29]. Integrins, including leukocyte function-associated antigen-1 (CD11a/CD18) expressed on all leukocytes and macrophage-1 (MAC-1; CD11b/CD18) expressed on neutrophils and monocytes, are transmembrane glycoproteins and mediate leukocyte-endothelium interactions [30]. In acute cerebral ischemia, the upregulation of integrins facilitates a firm adherence of leukocytes to endothelial ICAM-1; the leukocytes subsequently penetrate the endothelial basement membrane into the brain parenchyma. Thus, the tight junctions (TJs) between endothelial cells of the BBB are disrupted and become more permeable, leading to leukocyte infiltration [30]. This evidence revealed that circulating leukocytes adhere to the damaged endothelium as early as 4 h and achieve the peak at approximately 12–48 h after ischemic brain injury. Meanwhile, reactive microglia, platelets, and infiltrating leukocytes further release IL-1*β*, IL-6, TNF-*α*, ROS, MCP-1, MIP-1*α*, IL-8, and MMPs (mainly MMP-9) that exacerbate ischemic injury in MCAo [3, 5, 6].

### 3.2. The Effects and Mechanisms of TCMs on Inhibiting Leukocyte Infiltration in In Vivo Models of Cerebral Ischemia

Lu et al. reported that emodin, an active component of the rhizome of* Rheum palmatum* L. (Da Huang), effectively reduces the cerebral infarct size 6 h after MCAo. The neuroprotective effects of emodin can be attributed to ICAM-1 downregulation in the ischemic area in the early phase of cerebral ischemia [31]. Ferulic acid (FA), a major active compound in both* Angelica sinensis (Oliv.)* Diels (Dang Gui) and* Ligusicum chuanxiong* Hort. (Chuan Xiong), effectively reduces the cerebral infarct area and ameliorates the neurological deficit at 1.5 h of reperfusion and 24 h of reperfusion. FA exerts anti-inflammatory effects against cerebral I/R injury, at least partially, by inhibiting ICAM-1, mRNA, and Mac-1 mRNA expression in the ischemic striatum 2 h after reperfusion [32, 33]. Bu-yang Huan-wu decoction (BHD), composed of* Astragalus membranaceus* Bunge (Huang Qi),* A. sinensis (Oliv.)* Diels (Dang Gui),* Paeonia lactiflora* Pall (Shao Yao),* L. chuanxiong* (Chuan Xiong),* Prunus persica* (L.) Batsch (Tao Ren),* Carthamus tinctorius* L. (Hong Hua), and* Pheretima aspergillum* (Di Long), effectively ameliorates cerebral infarction and improves neurological deficits 24 h after transient MCAo. The effects of BHD against cerebral ischemic injury are partially attributed to the inhibition of CD11b (a marker of leukocyte and monocyte activation) expression in the ischemic area [34]. Persimmon leaf flavonoid isolated from* Diospyros kaki* L.F (Shi Zhi Ye) exerts anti-inflammatory effects by downregulating ICAM-1 expression in the ischemic area 2 h after cerebral ischemia and 24 h after reperfusion [35]. Liu et al. demonstrated that the oral administration of* Cordyceps sinensis* (Dong Chong Xia Cao) mycelium exerts neuroprotective effects against cerebral I/R injury by downregulating ICAM-1, IL-1*β*, and TNF-*α* expression and infiltrating polymorphonuclear leukocytes (PMNs) in the ischemic area 2 h after ischemia and 22 h after reperfusion [12]. The oral pretreatment with a herb formula, FuLing-BaiZhu-DangGui (FBD), composed of* Poria cocos* (Fu Ling),* Atractylodes macrocephala* (Bai Zhu), and* A. sinensis*, protects against cerebral I/R injury partially by inhibiting PMNs infiltration in the ischemic area 24 h after transient forebrain ischemia in mice. The anti-inflammatory properties of FBD can be further attributed to IL-1*β*, TNF-*α*, and IL-8 downregulation during the acute phase of cerebral ischemia [36]. Kong et al. reported that Borneol (Bing Pian), the resin of* Dryobalanops aromatica Gaertn*. F., effectively improves neurological deficits 24 h after forebrain ischemia. The beneficial effects of Borneol involve the reduction of leukocyte infiltration and ICAM-1 expression in the ischemic area [37].

These results indicate that TCMs provide beneficial effects against leukocyte infiltration mainly by downregulating ICAM-1 expression and activated leukocyte-induced cytokines, such as IL-1*β*, TNF-*α*, and IL-8, in the ischemic lesion in the acute phase of cerebral ischemia (Figure 1 and Table 2).

## 4. TCM-Mediated Stabilization of Blood-Brain Barrier Integrity

### 4.1. Blood-Brain Barrier Disruption during Cerebral Ischemia

Under normal conditions, leukocyte recruitment across the BBB into the brain parenchyma contributes to the maintenance of the central nervous system immune privilege [9]. The BBB comprising endothelial cells, the basement membrane, the astrocyte end-feet, and pericytes provides a highly selective permeability barrier that separates the blood cells from the brain interstitial fluid and maintains brain homeostasis [38]. However, during the acute phase of cerebral ischemia, the infiltrated leukocytes and reactive microglia synthesize and secrete MMPs (mainly MMP-2 and MMP-9) and ROS, thus increasing BBB permeability [72, 73]. Previous studies have reported that MMP-9 activation is initiated as early as 4 h, which reaches the maximum level at 24 h and persists for at least 5 d after cerebral ischemia [74, 75], whereas activated MMP-2 reaches the highest level 5 d after MCAo [75]. Active MMPs disrupt BBB integrity by degrading the extracellular matrix and TJs in endothelial cells and result in vascular and BBB leakage. The BBB disruption facilitates the entry of circulating leukocytes and intravascular fluid into the brain, which cause vasogenic edema and hemorrhagic transformation, leading to the exacerbation of cerebral infarction [6, 9, 76]. TJs, including claudin-5, occludin, and zonula occludens- (ZO-) 1, play a pivotal role in maintaining the structural and functional integrity of the BBB [77]. Previous studies have reported that decreased claudin-5, occludin, and ZO-1 expression is closely related to BBB disruption and ischemic brain edema formation [77, 78]. Thus, MMPs, claudin-5, occludin, and ZO-1 could present the potential targets for pharmacological intervention to stabilize BBB integrity in cerebral ischemic injury and the regulation of their activity may yield therapeutic effects.

### 4.2. The Effects and Mechanisms of TCMs on Ameliorating Blood-Brain Barrier Disruption in In Vivo Models of Cerebral Ischemia

Posttreatment methylophiopogonanone- (MO-) A, an active compound isolated from* Ophiopogon japonicus* (Mai Men Dong), effectively reduces the infarct volume and brain edema and improves neurological deficits 7 d after transient MCAo. The results indicate that MO-A protects against cerebral I/R injury mainly through its property to ameliorate BBB disruption through MMP-9 downregulation, and claudin-3 and claudin-5 upregulation in the ischemic cortex [38]. Tan et al. reported that pretreatment with ligustrazine, an active ingredient of* L. wallichii* Franchat (Chuan Xiong), effectively preserves BBB integrity by downregulating MMP-9 expression and upregulating claudin-5 and occludin expression in the ischemic area in a rat model of focal cerebral I/R injury [39]. Levo-tetrahydropalmatine (l-THP), a major active ingredient of* Rhizoma corydalis* (Yan Hu Suo), protects against cerebral I/R-induced BBB injury at 1.5 h of ischemia and 24 h of reperfusion. The protective effect of l-THP can be partially attributed to MMP-2 and MMP-9 downregulation and claudin-5, occludin, and ZO-1 upregulation in the ischemic area [40].

From these results, we conclude that MMP-9 downregulation and claudin-5, occludin, and ZO-1 upregulation are the potential effects of TCMs on the stabilization of BBB integrity to ameliorate inflammatory responses in the ischemic area during the acute and subacute phases of cerebral I/R injury (Figure 1 and Table 3).

## 5. TCM-Mediated Regulation of Proinflammatory Mediator Release

### 5.1. Toll-Like Receptor Stimulation on Microglia/Macrophages during Cerebral Ischemia

In the ischemic core, active microglia are indistinguishable from blood-derived macrophages, and the microglia/macrophages are apparent 3.5–12 h after transient focal cerebral ischemia [79]. Subsequently, active microglia/macrophages are distributed in the entire middle cerebral artery territory at 22–24 h and maintained for up to 1 week after cerebral ischemia [6]. During cerebral ischemic insult, the dying cells release DAMPs, including heat shock proteins (HSPs), *β*-amyloid, hyaluronan, high mobility group box 1 (HMGB1), heparin sulfate, and ATP, thus stimulating TLRs, which are expressed on microglia/macrophages. Thereafter, the microglia/macrophages transform into M1 and M2 phenotypes upon stimulation and secrete various cytokines in response to ischemic injury [73, 80, 81]. TLRs are pivotal components in the innate immune system, and TLRs (mainly TLR2 and TLR4) stimulation in microglia/macrophages and T-lymphocytes also exerts strong regulatory effects on postischemic inflammatory responses [2, 73]. During cerebral ischemic injury, TLRs facilitate cytokine and chemokine release and trigger transcription factor activation by activating intercellular signaling pathways. According to recruitment of specific adaptors, TLR signaling can be classified into either myeloid differentiation primary response gene 88- (MyD88-) dependent or independent pathways [81]. The binding of HSPs, such as HSP60 and HSP70, or HMGB1 with TLR2 and TLR4 initiate the expression of nuclear factor- (NF-) *κ*B, TNF-*α*, IL-1*β*, IL-6, inducible nitric oxide synthase (iNOS), and ICAM-1 by activating the MyD88-dependent signaling pathway and aggravating cerebral ischemic injury [82]. TLR2 and TLR4 levels markedly increase in the ischemic brain at 6 h, peak at 24 h, and decline at 72 h after MCAo [83].

### 5.2. The Effects and Mechanisms of TCMs on Suppressing Toll-Like Receptor Stimulation in In Vivo Models of Cerebral Ischemia

Zhou et al. reported that the anti-infarct effects of puerarin, a major isoflavonoid in* Radix puerariae* (Ge Gen), can be attributed to the downregulation of TLR4/MyD88/NF-*κ*B/TNF-*α* signaling in the ischemic region 24 h after transient MCAo [41]. TMP exerts anti-inflammatory effects against neutrophil activation 3 d after permanent MCAo. The beneficial effects of TMP can be partially attributed to HMGB1 and TLR4 downregulation in the ischemic cortex [42].

### 5.3. M1 Microglia/Macrophages Releasing Proinflammatory Mediators during Cerebral Ischemia

The M1 microglia/macrophages produce proinflammatory cytokines, including TNF-*α*, IL-1*β*, IL-6, IL-8, IL-12, IL-18, IL-20, and IFN-*γ*, whereas the M2 microglia/macrophages release anti-inflammatory cytokines, including IL-4, IL-10, IL-13, and TGF-*β* [1]. TNF-*α* is a pleiotropic cytokine that possesses both neurotoxic and neuroprotective properties. In the early phase of the inflammatory response, TNF-*α* binds to TNF receptor 1 (TNFR1) and contributes to the detrimental effects, such as promoting BBB disruption, vasogenic edema, leukocyte infiltration, and endothelial cell apoptosis [6, 84, 85]. Furthermore, TNF-*α*/TNFR1 activates NF-*κ*B signaling, which regulates the expression of cytokines; chemokines; adhesion molecules; and inducible enzymes, namely, iNOS and cyclooxygenase-2 (COX-2), thus exacerbating cerebral ischemic injury [6]. By contrast, in the late phase of postischemic inflammation, TNF-*α*/TNFR2 facilitates neuroprotection, synaptic plasticity, and tissue repair [6, 86]. TNF-*α* becomes predominant in the ischemic lesion 24–48 h after MCAo [87]. IL-1, including IL-1*α* and IL-1*β*, can bind to IL-1 receptor type 1 (IL-1R1) and majorly contribute to the exacerbation of ischemic brain injury [88]. IL-1*β* has been clearly implicated in the pathogenesis of cerebral ischemia [6]. Inactive proIL-1*β* is converted to biologically active IL-1*β* by an IL-1*β*-converting enzyme, which belongs to the cysteine protease family. IL-1*β* is initially upregulated at 1–3 h and peaks at 12–24 h after ischemic injury. The cytotoxic actions of IL-1*β* include facilitating the activation of microglia, infiltration of leukocytes, and production of other cytokines such as IL-6 [6, 89]. Conversely, IL-1 receptor antagonist, a member of the IL-1 family, binds to IL-1R1 and subsequently blocks the detrimental actions of IL-1, exerting neuroprotection in cerebral ischemia process [90]. The role of IL-6 in cerebral ischemia remains controversial. Some studies have reported that IL-6 aggravates cerebral infarction [91, 92], whereas other studies have reported the beneficial effects of IL-6 in preventing damaged neuron from undergoing apoptosis and promoting neuronal survival after cerebral ischemia [93, 94]. Moreover, IL-6 is predominantly expressed in the ischemic area 24–48 h following cerebral I/R injury [87]. IL-8, IL-12, IL-18, and IL-20 play a pivotal role in promoting cerebral ischemic injury [7, 16, 95, 96]. IL-18 is initiated within 24–48 h and peaks at 6 d in the ischemic region after cerebral ischemia [97]. IFN-*γ* contributes to the exacerbation of cerebral ischemia by increasing ischemia-induced glutamate release [98].

### 5.4. The Effects and Mechanisms of TCMs on Downregulating Proinflammatory Mediators in In Vivo Models of Cerebral Ischemia

Notoginseng saponins isolated from the root of* Panax notoginseng* (San Qi) provide beneficial effects against cerebral I/R injury partially through IL-1*β* mRNA downregulation in the ischemic area after 22 h of reperfusion [43]. Chang et al. reported that pretreatment with puerarin effectively reduces the cerebral infarct size and neurobehavioral deficits 24 h after MCAo. The anti-infarct effect of puerarin is, at least partially, because of the inhibition of TNF-*α* and iNOS expression in the ischemic area [44]. Li et al. explored the effect of osthole, a major active ingredient in* Cnidium monnieri* (L.) Gusson (She Chuang Zi), on acute cerebral I/R injury and reported that pretreatment with osthole markedly reduces the brain infarct volume and ameliorates neurological scores 24 h after MCAo. The neuroprotective effects of osthole are accompanied by the downregulation of proinflammatory mediators, including TNF-*α*, IL-1*β*, COX-2, and iNOS, expressed in the ischemic cortex [45]. The caffeic acid ester (Caf) fraction from* Erigeron breviscapus* (Deng Zhan Hua) significantly reduces the cerebral infarct volume and improves neurobehavioral performance at 1 h of ischemia and 24 h of reperfusion. The inhibition of iNOS, TNF-*α*, and IL-1*β* mRNA expression is one of the mechanisms underlying the neuroprotective effects of Caf against cerebral infarction [46]. Pretreatment with arctigenin, an active agent from* Arctium lappa* (Nu Bang Zi), effectively inhibits microglial activation and subsequently downregulates TNF-*α* and IL-1*β* expression in the penumbra region 24 h after transient MCAo [47]. Lee et al. reported that schisandrin B isolated from* Fructus schisandrae* (Wu Wei Zi) markedly reduces the cerebral infarct size and neurological deficits 24 h after transient focal cerebral ischemia. The anti-inflammatory effect of schisandrin B involves the inhibition of TNF-*α*, IL-1*β*, MMP-2, and MMP-9 expression and suppression of microglial activation in the ischemic area [48]. Posttreatment with asiaticoside, an active compound isolated from* Centella asiatica* (L.) (Ji Xue Cao), attenuates memory deficits by suppressing iNOS, TNF-*α*, IL-1*β*, and IL-6 expression in the hippocampus 7 d after transient bilateral common carotid artery occlusion [49]. Chen et al. reported that posttreatment with magnolol, an active ingredient of* Magnolia officinalis* (Hou Pu), ameliorates cerebral infarction partially by dose-dependently inhibiting iNOS, TNF-*α*, IL-1*β*, and IL-6 expression in the ischemic area 24 h after transient global ischemia [50]. Posttreatment with danhong, extracted from Radix* salviae miltiorrhizae* (Dan Shen) and* Flos carthami* (Hong Hua), exerts beneficial effects in cerebral I/R injury, at least partially, through dose-dependent IL-1*β* and TNF-*α* downregulation in the ischemia area at 1.5 h of ischemia and 14 d of reperfusion [51]. Gastrodin, an active constituent of* Gastrodia elata* Blume (Tian Ma), exerts an initial anti-inflammatory effect by suppressing TNF-*α* and IL-1*β* expression in the ischemic hemispheres 6 h after cerebral I/R injury [52].

### 5.5. The Effects and Mechanisms of TCMs on Regulating Anti-Inflammatory Cytokines in In Vivo Models of Cerebral Ischemia

IL-4, IL-10, IL-13, and TGF-*β* reduce microglia/macrophages-induced proinflammatory cytokines, such as IL-8 [99]. Moreover, IL-4 promotes long-term recovery after ischemic stroke [100]. IL-10 can inhibit IL-1 and TNF-*α* expression [55] and prevent the downregulation of the antiapoptotic protein Bcl-2 expressed in ischemic brain lesion [101]. IL-4 mRNA generates as early as 1 h, reaches a peak at 3–24 h, and gradually declines 2 d following ischemic stroke [102]. Pretreatment with danshen, an aqueous extract of the root and rhizome of* Salvia miltiorrhiza* Bunge (Dan Shen), protects against cerebral I/R injury in association with decreased IL-10 and TNF-*α* mRNA and protein expression in the ischemic area 24 h after transient MCAO [53]. Guizhi fuling capsules, composed of* Cinnamomum cassia* Blume (Gui Zhi),* P. lactiflora* Pall (Shao Yao),* P. suffruticosa* Andrews (Mu Dan Pi),* P. persica* Batsch (Tao Ren), and* Poria cocos* Wolf (Fu Ling), protect against cerebral infarction through TNF-*α* and IL-1*β* mRNA and protein downregulation and IL-10 and IL-10 receptor (IL-10R) mRNA and protein upregulation in the ischemia area after 2 h of ischemia and 24 h of reperfusion [54]. Zhang et al. also reported that the Gualou Guizhi decoction composed of* Trichosanthis *radix (Tian Hua Fen),* Ramulus cinnamomi* (Gui Zhi),* P. lactiflora *(Shao Yao),* Glycyrrhiza* (Gan Zao),* Zingiber officinale* Roscoe (Sheng Jiang), and* Fructus jujubae* (Da Zao) exerts neuroprotection against cerebral I/R injury through IL-1, TNF-*α*, and NF-*κ*B downregulation and IL-10 upregulation in the ischemic area in the subacute phase (7 d) after transient MCAo [55].

### 5.6. The Effects and Mechanisms of TCMs on Downregulating Proinflammatory Enzymes in In Vivo Models of Cerebral Ischemia

COX-2 and 5-lipoxygenase (5-LO) are rate-limiting enzymes that convert arachidonic acid to prostaglandins and leukotrienes [57]. In the delayed phase of cerebral ischemia, microglia/macrophages produce 5-LO, which converts arachidonic acid to leukotrienes. Leukotrienes are potent inflammatory mediators that trigger chemotaxis of leukocytes and BBB damage and subsequently cause vasogenic edema, thus exacerbating cerebral ischemia [103]. COX-2 and 5-LO expression is markedly enhanced in the ischemic cortex 24 h after cerebral I/R injury [57]. Guo et al. explored the anti-infarct effect of paeoniflorin (PF), the principle component of* P. radix* (Shao Yao), in the subacute phase of cerebral I/R injury and reported that PF protects against cerebral infarction mainly through TNF-*α*, IL-1*β*, iNOS, COX-2, and 5-LO downregulation in the ischemic area 14 d after reperfusion [56]. Chen et al. reported that pretreatment with PF effectively ameliorates the cerebral infarct volume and neurological deficits 24 h after reperfusion in a model of pharmacological preconditioning. The neuroprotective effects of PF against cerebral I/R injury are partially related to the inhibition of COX-2, 5-LO, and iNOS expression in the ischemic lesion [57].

According to the aforementioned studies, proinflammatory mediators, such as TLR4, TNF-*α*, IL-1*β*, IL-6, IL-18, COX-2, and 5-LO, are predominately expressed in the ischemic area 24 h after MCAo. TCMs effectively ameliorate cerebral I/R injury by downregulating TLR4, TNF-*α*, IL-1*β*, IL-6, iNOS, COX-2, and 5-LO expression and upregulating IL-10 expression in the ischemic area during the acute and subacute phases of cerebral ischemia (Figure 1 and Table 4).

## 6. TCM-Mediated Regulation of Transcription Factor Activation

### 6.1. NF-*κ*B Activation during Cerebral Ischemia

NF-*κ*B is a classic transcription factor and plays a crucial role in the regulation of hundreds of genes involved in cell survival and death [104]. Thus, NF-*κ*B can be activated via several intracellular signaling pathways associated with host defense, inflammation, and apoptosis [58]. In the brain, NF-*κ*B regulates the expression of different sets of genes, such as antiapoptotic, proapoptotic, and proinflammatory genes, thereby playing a dual role in neuronal survival and death [63]. The NF-*κ*B family includes five members, namely, p65 (RelA), RelB, c-Rel, p50/p105 (NF-*κ*B1), and p52/p100 (NF-*κ*B2), which form various homo- and heterodimeric complexes [2]. The most common form of NF-*κ*B is the p65/p50 heterodimer [6]. Under an unstimulated condition, the inhibitor of NF-*κ*B proteins (I*κ*Bs), including mainly I*κ*B*α*, I*κ*B*β*, and I*κ*B*ε*, retain inactive NF-*κ*B dimmers in the cytosol, whereas in response to various extracellular stimuli, including infection, proinflammatory cytokines, and antigen receptor engagement, the activated I*κ*B kinase complexes phosphorylate I*κ*B proteins, resulting in their ubiquitination and proteasomal degradation and consequently inducing the release of NF-*κ*B for nuclear translocation and the activation of target gene transcription [6, 105]. During cerebral ischemia, the activated NF-*κ*B dimers are subsequently translocated into the nucleus where they selectively bind to specific DNA sequences called *κ*B sites; promoter domains present a large number of proinflammatory genes and subsequently cause TNF-*α*, IL-1*β*, IL-6, ICAM-1, PGE2, COX-2, and iNOS translation [6, 105, 106]. NF-*κ*B activators include some proinflammatory cytokines, such as TNF-*α* and IL-1*β*, whose genes are regulated by NF-*κ*B itself, inducing a positive feedback loop and resulting in the amplification of the inflammatory response and exacerbation of cerebral ischemic insults [107]. Previous studies have indicated that NF-*κ*B activation occurs as early as 1 h, reaches a peak at 6 h, and sustains for at least 72 h in the cerebral ischemic area in rats [62, 107].

### 6.2. The Effects and Mechanisms of TCMs on Downregulating NF-*κ*B Activation in In Vivo Models of Cerebral Ischemia

Wogonin, a flavonoid derived from* Scutellaria baicalensis* Georgi (Huang Qin), exerts neuroprotective effects by inhibiting the inflammatory activation of microglia in an in vitro cell culture model. The anti-inflammatory effects of wogonin are partially attributed to the downregulation of NF-*κ*B-mediated iNOS and TNF-*α* expression in the ischemic hippocampal CA1 area in transient global cerebral ischemia in rats [58]. Tanshinone IIA (Ts IIA) and IIB, the key compounds of* S. miltiorrhiza* Bunge, effectively reduce the cerebral infarct volume and improve the neurological function 24 h after transient MCAo [59]. Dong et al. further reported that pretreatment with Ts IIA protects against cerebral infarction partially associated with the reduction of ROS-mediated NF-*κ*B activation, leading to the inhibition of iNOS expression in the ischemic area 24 h after permanent MCAo [60]. Silymarin, a bioactive component isolated from* Silybum marianum* (Shui Fei Ji), provides neuroprotection against cerebral I/R injury by inhibiting oxidative and nitrosative stress in the ischemic area 24 h after cerebral ischemia. The antioxidative and antinitrosative effects of silymarin are partially attributed to the reduction of NF-*κ*B-mediated iNOS, COX-2, ICAM-1, TNF-*α*, and IL-1*β* expression in the injured tissues [61]. In addition, Guan et al. reported that ruscogenin, a major effective compound isolated from* O. japonicus* Ker-Gawl, ameliorates cerebral I/R injury through the downregulation of NF-*κ*B target genes, including* ICAM-1*,* iNOS*,* COX-2*,* TNF-α*, and* IL-1β*, in the ischemic area 24 h after reperfusion [62]. Hydroxysafflor yellow A, a major active component of* C. tinctorius* L. (Hong Hua), reduces cerebral infarction by suppressing cytosolic NF-*κ*Bp65 translocation to the nucleus and subsequently downregulates NF-*κ*B-mediated TNF-*α*, IL-1*β*, and IL-6 expression in the ischemic area 24 h after permanent MCAo [63]. Chern et al. reported that 2-methoxystypandrone (2-MS), a major active component of* Polygonum cuspidatum* (Hu Zhang), attenuates the brain infarct size and improves the neurological function, at least partially, by preventing I*κ*B*α* degradation and a reducing NF-*κ*B-mediated iNOS and COX-2 expression in the peri-infarct cortex 24 h after transient MCAo. The anti-inflammatory effects of 2-MS can further contribute toward the preserving BBB integrity [64]. Previous studies have indicated that p38 mitogen-activated protein kinase (MAPK), one of the MAPK family members, upregulates NF-*κ*B expression (p38 MAPK/NF-*κ*B signaling) and subsequently causes the transcription of genes encoding proinflammatory cytokines, resulting in the exacerbation of cerebral infarction in the acute phase of transient MCAo [108–110]. In addition, activated p38 MAPK occurs in the ischemic area as early as 2 h and reaches a peak 24–48 h after reperfusion [110]. Piperlonguminine from* Piper longum* (Bi Bo) alkaloids protects against cerebral ischemic injury by inhibiting the activation of p38 MAPK/NF-*κ*B signaling cascade in the ischemic region 24 h after permanent MCAo [65].

### 6.3. The Effects and Mechanisms of TCMs on Upregulating Peroxisome Proliferator-Activated Receptor Activation in In Vivo Models of Cerebral Ischemia

Peroxisome proliferator-activated receptors (PPARs) include PPAR*α*, PPAR*γ*, and PPAR*δ*/*β* isoforms, which are members of the nuclear receptor superfamily and represent ligand-activated transcription factors. PPAR*γ* is predominantly expressed in the central nervous system and binds to peroxisome proliferator response elements to regulate its target gene expression [66]. During cerebral ischemia, PPAR*γ* is detected in the peri-infarct area as early as 4 h and is sustained for at least 14 d after ischemia [111]. PPAR*γ* exerts neuroprotective effects against inflammatory mediators to initiate responses by inhibiting the activation of NF-*κ*B signaling in the ischemic area after focal cerebral ischemia [66, 111]. Liu et al. reported that pretreatment with curcumin, a natural polyphenolic component of* curcuma longa* (Jiang Huang), markedly reduces the cerebral infarct volume by activating PPAR*γ* signaling in the ischemic cortex 24 h after reperfusion. The effects of curcumin on the regulation of PPAR*γ* signaling further contribute to the downregulation of NF-*κ*Bp65-mediated TNF-*α*, IL-1*β*, iNOS, PGE2, and COX-2 expression [66]. Another study revealed that pretreatment with icariin, a natural flavonoid compound extracted from* Epimedium brevicornum maxim* (Yin Yang Huo), protects against cerebral I/R injury by activating PPAR*α* and PPAR*γ* and subsequently suppressing NF-*κ*Bp65-mediated IL-1*β* expression in the ischemic cortex at 2 h of ischemia and 24 h of reperfusion [67].

### 6.4. The Effects and Mechanisms of TCMs on Regulating Signal Transducer and Activator of Transcription Activation in In Vivo Models of Cerebral Ischemia

Signal transducer and activator of transcription (STAT) proteins are a family of transcription factors comprising seven members, namely, STAT1, STAT2, STAT3, STAT4, STAT5a, STAT5b, and STAT6 [112]. Some controversy exists on whether STAT signaling is neuroprotective or neurotoxic in cerebral ischemic injury [113]. Growing evidence has revealed that cytokines induce the activation of the receptor-associated Janus kinases (JAKs), including JAK1, JAK2, JAK3, and tyrosine kinase 2, which consequently activate STAT proteins; the activated STAT proteins undergo dimerization and translocation to the nucleus, thereby regulating the expression of proapoptotic and proinflammatory genes [114, 115]. Among STAT protein isoforms, STAT3 is the most-conserved isoform [68]. In the transient cerebral ischemic rat model, STAT3 is activated as early as 30 min and sustained 24 h after reperfusion, and activated STAT3 triggers cerebral I/R injury by amplifying inflammatory responses [116]. However, other studies have reported that activated STAT3 signaling provides neuroprotection by upregulating Bcl-2 and vascular endothelial growth factor expression in the peri-infarct area after transient cerebral ischemia [113, 117]. Kaempferol-3-O-rutinoside (KRS) and kaempferol-3-O-glucoside (KGS) are also the active components of* C. tinctorius* L. Both KRS and KGS markedly reduce the cerebral infarct volume at least partially associated with the inhibition of STAT3 and NF-*κ*Bp65 activation, and subsequently, proinflammatory mediators (TNF-*α*, IL-1*β*, iNOS, MMP-9, and ICAM-1) production in the cortical penumbra 24 h after transient MCAo [68]. Astragaloside IV [the active component of* Astragalus* (Huang Qi)] combined with Ginsenoside Rg1, Ginsenoside Rb1, and Notoginsenoside R1 (the active components of *P*. notoginseng) effectively restores cell survival partially related to the inhibition of JAK1/STAT1 and NF-*κ*B signaling and consequently suppresses TNF-*α*, IL-1*β*, and ICAM-1 mRNA expression in the ischemic area 24 h after transient global cerebral ischemia [69]. By contrast, Li et al. reported that curcumin reduces cerebral infarction and attenuates neurological deficits by activating JAK2/STAT3 signaling and downregulating IL-1*β* and IL-8 in the injured region after 24 h of reperfusion [70].

### 6.5. The Effects and Mechanisms of TCMs on Regulating Nuclear Factor-Erythroid 2-Related Factor 2/Heme Oxygenase-1 and c-Jun N-Terminal Kinase/c-Jun/Activating Protein-1 Signaling in an In Vivo Model of Cerebral Ischemia

Nuclear factor-erythroid 2-related factor 2 (Nrf2), a potent cytoprotective transcription factor, induces the expression of genes encoding antioxidant and anti-inflammatory proteins [118]. Under stress, Nrf2 dissociates from its cytoplasmic inhibitory protein Kelch-like ECH-associated protein 1 and translocates into the nucleus, where it binds to an antioxidant response element and regulates target genes, including heme oxygenase-1 (HO-1). The Nrf2/HO-1 signaling pathway attenuates inflammatory responses in cerebral ischemia [71]. During transient focal cerebral ischemia, Nrf2 and HO-1 occur in the ischemic cortex as early as 6 h, up to a maximum 48 h and decline 72 h after cerebral I/R [119]. The activation of c-Jun N-terminal kinase (JNK), one of the MAPK family members, signaling plays a central role in ischemia-induced neuroinflammation. When stimulated, activated JNK translocates into the nucleus and phosphorylates c-Jun, the major component of activating protein- (AP-) 1, which comprises c-Jun and c-Fos proteins, leading to the expression of target genes encoding proinflammatory mediators. JNK/AP-1 signaling amplifies the inflammatory response during cerebral ischemia [71]. JNK/c-Jun/AP-1 signaling factors are predominantly expressed in the ischemic area 2 h after cerebral ischemia [120]. Kao et al. reported that TMP effectively reduces cerebral infarction by inhibiting microglia/macrophages activation in the ischemic cortex 72 h after permanent MCAo. The anti-inflammatory effects of TMP can be further attributed to the upregulation of Nrf2/HO-1 signaling and downregulation of JNK/c-Jun/AP-1 signaling in the ischemic cortex [71].

**Figure 2:**
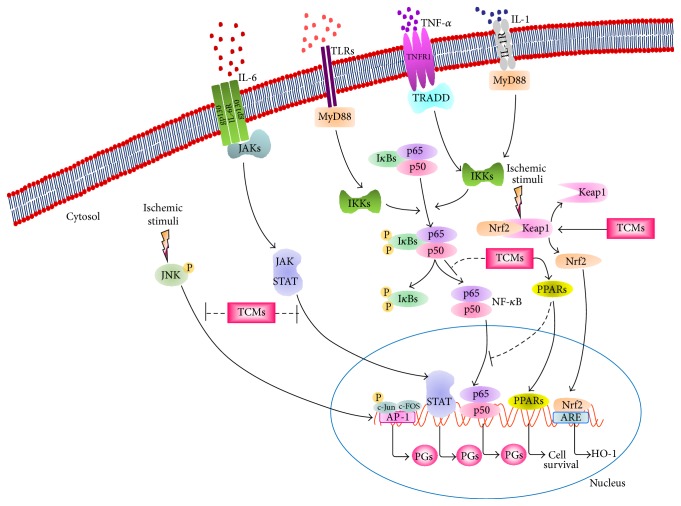
Schematic representation of the anti-inflammatory effects of traditional Chinese medicines through the regulation of transcription factors in the inflammatory cascade after cerebral ischemia. JAKs, Janus kinases; MyD88, myeloid differentiation primary response gene 88; TRADD, tumor necrosis factor receptor type 1-associated death domain; I*κ*Bs, inhibitor of NF-*κ*B proteins; IKKs, I*κ*B kinases; Nrf2, nuclear factor-erythroid 2-related factor 2; Keap1, Kelch-like ECH-associated protein 1; PPARs, peroxisome proliferator-activated receptors; STAT, signal transducer and activator of transcription; JNK, c-Jun N-terminal kinase; AP-1, activating protein-1; ARE, antioxidant response element; HO-1, heme oxygenase-1; PGs, proinflammatory genes. Thick solid lines with arrowheads indicate activation, and thin dotted lines indicate inhibition.

According to the aforementioned studies, NF-*κ*B and JNK/AP-1 signaling induced in the ischemic brain may amplify inflammatory responses, whereas PPARs and Nrf2/HO-1 signaling are considered to prevent postischemic inflammation and yield potent effects against cerebral ischemic injury. JAK/STAT signaling plays a dual role in the regulation of proinflammatory mediators depending on the experimental models of brain ischemia. TCMs protect against cerebral ischemic injury by inhibiting deleterious transcription factors (NF-*κ*B, JAK/STAT, and JNK/AP-1), activating neuroprotective transcription factors (PPARs and Nrf2/HO-1) and consequently regulating the expression of transcription factor-mediated proinflammatory genes (TNF-*α*, IL-1*β*, IL-6, IL-8, iNOS, COX-2, PGE2, MMP-9, and ICAM-1) in the ischemic area in the early stage (24–72 h) of cerebral ischemia (Figure 2 and Table 5).

## 7. Conclusions

After the onset of cerebral ischemia, resident microglia are rapidly activated (within a few minutes) and subsequently produce large amounts of cytokines, chemokines, and ROS, thus causing the initial ischemic injury. TCMs can exert neuroprotective effects against the initial ischemic injury by rapidly downregulating ischemia-induced microglial activation and microglia-mediated proinflammatory cytokine production in the ischemic region. The microglial production of proinflammatory mediators subsequently increase adhesion molecule expression, facilitate leukocyte-endothelial cell interactions, and activated leukocytes that penetrate the endothelial cell barrier into the brain parenchyma (as early as 4 h after the ischemic onset). The infiltrating leukocytes further release inflammatory mediators in the ischemic lesion, thus exacerbating ischemic injury. TCMs can effectively attenuate leukocyte infiltration by inhibiting ICAM-1 and activated leukocyte-induced cytokine expression in the ischemic region in the early phase of cerebral ischemia. Meanwhile, infiltrated leukocytes and activated microglia secrete MMPs, which cause the disruption of BBB integrity, worsening cerebral infarction. TCMs effectively inhibit MMPs expression and stabilize BBB integrity to ameliorate cerebral infarction. Increased TLRs stimulation in microglia/macrophages (activated microglia and recruited leukocytes) by the activation of intercellular signaling pathways robustly secretes various proinflammatory mediators (cytokines and enzymes) in the ischemic region 6–24 h after ischemia. TCMs can timely rescue the injured neurons by downregulating proinflammatory receptors (TLRs), cytokines, and enzymes and upregulating anti-inflammatory cytokine expression in the ischemic lesion. The proinflammatory transcription factors are subsequently activated by the secreted cytokines, whose genes are regulated by these transcription factors themselves, thus inducing a positive feedback loop, in which the inflammatory response is amplified and secondary brain injury is exacerbated 24–72 h after cerebral ischemia. TCMs protect against inflammatory response-induced secondary brain injury by inhibiting deleterious transcription factors, activating neuroprotective transcription factors, and consequently regulating the expression of transcription factor-mediated proinflammatory genes in the ischemic area. Therefore, TCMs provide promising anti-inflammatory therapeutic strategies in the acute phase of cerebral ischemia. However, further studies are needed to elucidate the precise mechanisms of TCMs against inflammatory responses in the ischemic cascade after stroke.

## Figures and Tables

**Table 1:** TCMs downregulate microglial activation in the inflammatory cascade in ischemic stroke models.

TCMs	Isolated from the Chinese herb (Chinese name)	Anti-inflammatory actions	Models	References
Paeonol	Mu Dan	ED1↓, IL-1*β*↓	MCAo 1.5 h of ischemia followed by 24 h of reperfusion	[20]

Tetramethylpyrazine	Chuan Xiong	MPO↓, ED1↓, PGE2↓	MCAo 1.5 h of ischemia followed by 72 h of reperfusion	[21]

Tetramethylpyrazine	Chuan Xiong	MCP-1↓	MCAo 1.5 h of ischemia followed by 72 h of reperfusion	[22]

Andrographolide	Chuan Xin Lian	NF-*κ*Bp65↓, TNF-*α*↓, IL-1*β*↓, PGE2↓	Permanent MCAo 24 h of ischemia	[23]

Sophora japonica L	Huai Hua	ED1↓, IL-1*β*↓	MCAo 1.5 h of ischemia followed by 24 h of reperfusion	[24]

Isoflavones	Ge Gen	COX-2↓, GFAP↓, OX-42↓	MCAo 2 h of ischemia followed by 2 or 7 d of reperfusion	[25]

ED1, CD68 (macrophage marker); MPO, myeloperoxidase; PGE2, prostaglandin E2; MCP-1, monocyte chemoattractant protein-1; COX-2, cyclooxygenase-2; GFAP, glial fibrillary acidic protein; OX-42, CD11b (microglial activation marker).

**Table 2:** TCMs suppress leukocyte infiltration in the inflammatory cascade in ischemic stroke models.

TCMs	Isolated from the Chinese herb (Chinese name)	Anti-inflammatory actions	Models	References
Emodin	Da Huang	ICAM-1↓, TNF-*α*↓, IL-1*β*↓	MCAo 6 h of ischemia	[31]

Ferulic acid	Dang Gui or Chuan Xiong	ICAM-1↓, ICAM-1 mRNA↓, MPO↓, NF-*κ*Bp50↓,	MCAo 1.5 h of ischemia followed by 2 or 24 h of reperfusion	[32, 33]

Bu-yang Huan-wu decoction	Huang Qi, Dang Gui, Shao Yao, Chuan Xiong, Tao Ren, Hong Hua, Di Long	CD11b↓	MCAo 0.5 h of ischemia followed by 24 h of reperfusion	[34]

Persimmon leaf flavonoid	Shi Zhi Ye	ICAM-1↓	MCAo 2 h of ischemia followed by 24 h of reperfusion	[35]

Cordyceps sinensis	Dong Chong Xia Cao	ICAM-1↓, TNF-*α*↓, IL-1*β*↓, NF-*κ*Bp50↓, iNOS↓, COX-2↓	MCAo 2 h of ischemia followed by 22 h of reperfusion	[12]

FuLing-BaiZhu-DangGui	Fu Ling, Bai Zhu, Dang Gui	TNF-*α*↓, IL-1*β*↓, IL-8↓, MPO↓, NF-*κ*B↓	Repetitive BCCAo 10 min of ischemia (repeat 2 times) followed by 24 h of reperfusion	[36]

Borneol	Bing Pian	ICAM-1↓, TNF-*α*↓	MCAo 2 h of ischemia followed by 22 h of reperfusion	[37]

ICAM-1, intercellular adhesion molecule-1; BCCAo, bilateral common carotid artery occlusion.

**Table 3:** TCMs stabilize blood-brain barrier integrity in the inflammatory cascade in ischemic stroke models.

TCMs	Isolated from the Chinese herb (Chinese name)	Anti-inflammatory actions	Models	References
Methylophiopogonanone A	Mai Men Dong	MMP-9↓, claudin-3↑, claudin-5↑	MCAo 2 h of ischemia followed by 7 d of reperfusion	[38]

Ligustrazine	Chuan Xiong	MMP-9↓, claudin-5↑, occludin↑	MCAo 1.5 h of ischemia followed by 22.5 h of reperfusion	[39]

Levo-tetrahydropalmatine	Yan Hu Suo	MMP-2↓, MMP-9↓ claudin-5↑, occludin↑, ZO-1↑	MCAo 1.5 h of ischemia followed by 24 h of reperfusion	[40]

MMP-9, matrix metalloproteinase-9; ZO-1, zonula occludens-1.

**Table 4:** TCMs regulate the cytokine release in the inflammatory cascade in ischemic stroke models.

TCMs	Isolated from the Chinese herb (Chinese name)	Anti-inflammatory actions	Models	References
Puerarin	Ge Gen	TLR4↓, MyD88↓, NF-*κ*Bp65↓, TNF-*α*↓	MCAo 1.5 h of ischemia followed by 24 h of reperfusion	[41]

Tetramethylpyrazine	Chuan Xiong	TLR4↓, HMGB1↓, Nrf2↑, HO-1↑	Permanent MCAo 3 d of ischemia	[42]

Notoginseng	San Qi	IL-1*β*↓	MCAo 2 h of ischemia followed by 22 h of reperfusion	[43]

Puerarin	Ge Gen	TNF-*α*↓, iNOS↓	MCAo 1 h of ischemia followed by 24 h of reperfusion	[44]

Osthole	She Chuang Zi	TNF-*α*↓, IL-1*β*↓, COX-2↓, iNOS↓	Permanent MCAo 24 h of ischemia	[45]

Caffeic acid ester	Deng Zhan Hua	iNOS mRNA↓, TNF-*α* mRNA↓, IL-1*β* mRNA↓	MCAo 1 h of ischemia followed by 24 h of reperfusion	[46]

Arctigenin	Nu Bang Zi	TNF-*α*↓, IL-1*β*↓, OX-42↓	MCAo 2 h of ischemia followed by 24 h of reperfusion	[47]

Schisandrin B	Wu Wei Zi	TNF-*α*↓, IL-1*β*↓, MMP-2↓, MMP-9↓, OX-42↓	MCAo 2 h of ischemia followed by 24 h of reperfusion	[48]

Asiaticoside	Ji Xue Cao	iNOS↓, TNF-*α*↓, IL-1*β*↓, IL-6↓	BCCAo 10 min of ischemia (repeat 2 times) followed by 7 d of reperfusion	[49]

Magnolol	Hou Pu	iNOS↓, TNF-*α*↓, IL-1*β*↓, IL-6↓, NF-*κ*Bp65↓	BCCAo 1.5 h of ischemia followed by 24 h of reperfusion	[50]

Danhong injection	Dan Shen and Hong Hua	TNF-*α*↓, IL-1*β*↓	MCAo 1.5 h of ischemia followed by 14 d of reperfusion	[51]

Gastrodin	Tian Ma	TNF-*α*↓, IL-1*β*↓	MCAo 1 h of ischemia followed by 6 h of reperfusion	[52]

Danshen	Dan Shen	IL-10 mRNA↓, TNF-*α* mRNA↓, IL-10↓, TNF-*α*↓	MCAo 1 h of ischemia followed by 24 h of reperfusion	[53]

Guizhi fuling capsules	Gui Zhi, Shao Yao, Mu Dan, Tao Ren, Fu Ling	TNF-*α* mRNA↓, IL-1*β* mRNA↓, TNF-*α*↓, IL-1*β*↓, IL-10 mRNA↑, IL-10R mRNA↑, IL-10↑, IL-10R↑	MCAo 2 h of ischemia followed by 24 h of reperfusion	[54]

Gualou Guizhi decoction	Tian Hua Fen, Gui Zhi, Shao Yao, Gan Zao, Sheng Jiang, Da Zao	TNF-*α*↓, IL-1↓, NF-*κ*Bp65↓, IL-10↑	MCAo 2 h of ischemia followed by 7 d of reperfusion	[55]

Paeoniflorin	Shao Yao	TNF-*α*↓, IL-1*β*↓, iNOS↓, COX-2↓, 5-LO↓	MCAo 1.5 h of ischemia followed by 14 d of reperfusion	[56]

Paeoniflorin	Shao Yao	COX-2↓, 5-LO↓, iNOS↓	MCAo 1.5 h of ischemia followed by 24 h of reperfusion	[57]

TLR4, toll-like receptor 4; MyD88, myeloid differentiation primary response gene 88; HMGB1, high mobility group box 1; Nrf2, nuclear factor-erythroid 2-related factor 2; HO-1, heme oxygenase-1; iNOS, inducible nitric oxidase synthase; 5-LO, 5-lipoxygenase.

**Table 5:** TCMs regulate transcription factors in the inflammatory cascade in ischemic stroke models.

TCMs	Isolated from the Chinese herb (Chinese name)	Anti-inflammatory actions	Models	References
Wogonin	Huang Qin	NF-*κ*Bp65↓, iNOS↓, TNF-*α*↓	4-VO 10 min of ischemia followed by 7 d of reperfusion	[58]

Tanshinone IIA	Dan Shen	NF-*κ*Bp65↓, iNOS↓	MCAo 2 h of ischemia followed by 24 h of reperfusion Permanent MCAo 24 h of ischemia	[59, 60]

Silymarin	Shui Fei Ji	NF-*κ*Bp65↓, iNOS↓, COX-2↓, ICAM-1↓, IL-1*β*↓, MPO↓	MCAo 1 h of ischemia followed by 24 h of reperfusion	[61]

Ruscogenin	Mai Men Dong	NF-*κ*Bp65↓, ICAM-1↓, iNOS↓, COX-2↓, TNF-*α*↓, IL-1*β*↓	MCAo 1 h of ischemia followed by 24 h of reperfusion	[62]

Hydroxysafflor yellow A	Hong Hua	NF-*κ*Bp65↓, TNF-*α*↓, IL-1*β*↓, IL-6↓	Permanent MCAo 24 h of ischemia	[63]

2-Methoxystypandrone	Hu Zhang	NF-*κ*Bp65↓, I*κ*B*α*↑, iNOS↓, COX-2↓,	MCAo 40 min of ischemia followed by 24 h of reperfusion	[64]

Piperlonguminine	Bi Bo	NF-*κ*Bp65↓, p-p38 MAPK↓	Permanent MCAo 24 h of ischemia	[65]

Curcumin	Jiang Huang	PPAR*γ*↑, NF-*κ*Bp65↓, I*κ*B*α*↑, TNF-*α*↓, IL-1*β*↓, iNOS↓, PGE2↓, COX-2↓	MCAo 2 h of ischemia followed by 24 h of reperfusion	[66]

Icariin	Yin Yang Huo	PPAR*α*↑, PPAR*γ*↑, NF-*κ*Bp65↓, IL-1*β*↓	MCAo 2 h of ischemia followed by 24 h of reperfusion	[67]

Kaempferol-3-O-rutinoside and kaempferol-3-O-glucoside	Hong Hua	STAT3↓, NF-*κ*Bp65↓ TNF-*α*↓, IL-1*β*↓, iNOS↓, MMP-9↓, ICAM-1↓	MCAo 2 h of ischemia followed by 24 h of reperfusion	[68]

Astragaloside IV, ginsenoside Rg1, ginsenoside Rb1, notoginsenoside R1	Huang Qi and San Qi	JAK1↓, STAT1↓, NF-*κ*Bp65↓, p-I*κ*B*α*↓ TNF-*α* mRNA↓, IL-1*β* mRNA↓, ICAM-1 mRNA↓	BCCAo 20 min of ischemia followed by 24 h of reperfusion	[69]

Curcumin	Jiang Huang	JAK2↑, STAT3↑, IL-1*β*↓, IL-8↓	MCAo 1.5 h of ischemia followed by 24 h of reperfusion	[70]

Tetramethylpyrazine	Chuan Xiong	Nrf2↑, HO-1↑, MPO↓, p-c-Jun↓, p-JNK↓, AP-1↓	Permanent MCAo 72 h of ischemia	[71]

4-VO, 4-vessel occlusion; I*κ*B*α*, inhibitor of NF-*κ*B protein *α*; PPAR*γ*, peroxisome proliferator-activated receptor *γ*; STAT3, signal transducer and activator of transcription 3; JAK1, Janus kinase 1; AP-1, activating protein-1.
